# Improved stage-specific survival in screen-detected breast cancer in Denmark: a cohort study

**DOI:** 10.1093/jnci/djaf377

**Published:** 2026-02-19

**Authors:** Amy Tickle, Judith Offman, Bernard North, Susanne Fogh Jørgensen, Sisse Njor, Peter Sasieni

**Affiliations:** Comprehensive Cancer Centre, King’s College London, Guy’s Campus, London, United Kingdom; Wolfson Institute of Population Health, Queen Mary University of London, London, United Kingdom; Wolfson Institute of Population Health, Queen Mary University of London, London, United Kingdom; Department of Regional Health Research, University of Southern Denmark, Odense, Denmark; Research Unit for Screening and Epidemiology, Department for Biochemistry and Immunology, University Hospital of Southern Denmark, Vejle, Denmark; Department of Regional Health Research, University of Southern Denmark, Odense, Denmark; Research Unit for Screening and Epidemiology, Department for Biochemistry and Immunology, University Hospital of Southern Denmark, Vejle, Denmark; Department of Clinical Medicine, Aarhus University, Aarhus, Denmark; Wolfson Institute of Population Health, Queen Mary University of London, London, United Kingdom

**Keywords:** breast screening, mammography, organised breast screening programs, stage at diagnosis, stage-specific survival, net survival, stage 4, breast cancer, metastatic, metastatisis, late-stage, screen-detected, route to diagnosis, oligometastatic, excess mortality

## Abstract

**Background:**

This study examined whether breast cancer survival improvements with screening are explained solely by detection at early stages and whether mortality can be predicted using stage and diagnosis date alone.

**Methods:**

We compared stage-specific net survival between never-screened, symptomatic ever-screened (past attenders and interval cancers), and screen-detected breast cancer cases in Denmark using individual-level electronic health records from January 1, 2010 to December 31, 2022. Lifetables were generated from women without breast cancer, separately for never-screened and ever-screened women. Age-specific all-cause mortality rates were used to calculate excess mortality in women with breast cancer, which was then transformed into net survival.

**Results:**

Of 817 128 women, 32 827 had breast cancer, with 8% presenting as stage III or IV. Survival differences between symptomatic and screen-detected cases were minimal for stages I–III but reached 40% at stage IV, with 5-year net survival for stage IV screen-detected women (74.7%) resembling stage IIIc symptomatic survival in never-screened women (72.6%). Survival from stage IV breast cancer was strongly associated with treatment, with median survival (years) of 4.4-6.0 with surgery, 1.6-2.2 with nonsurgical treatment, and 0.03-0.13 with no treatment; 67% of screen-detected patients received surgery (compared with 23% of never-screened and 27% of symptomatic ever-screened).

**Conclusions:**

Greater survival in screen-detected stage IV cases suggests that breast cancer screening may not have come too late and deserves to be investigated further. Predicting breast cancer mortality using stage at diagnosis and stage-specific survival (without differentiating by route to diagnosis) will underestimate the impact of breast screening on mortality.

## Introduction

Organized breast cancer screening programs provide women with national access to 2-view, digital mammography, followed by diagnostic assessment for abnormal screens. The ages at which screening is offered varies between countries,^1-3^ while screening intervals range from 1-3 years (annually in the United States, biennially in Denmark, and triennially in the United Kingdom).

In Denmark, breast screening implementation became mandatory at county level from 2007, with national rollout being required by the end of 2009. Since then, biennial breast cancer screening has been offered to female residents aged 50-69 years. This service is administered separately by 5 administrative regions, with women with detected abnormalities being recommended for further diagnostic tests. All cancer treatment is free-of-charge in Denmark.

Both screening and assessment are monitored and evaluated using process and outcome measures to ensure successful program delivery.^4^ The latter type includes the rate of advanced breast cancer. The justification for looking at stage at diagnosis is that it is assumed to be independently predictive of breast cancer mortality, so that a reduction in advanced-stage disease will correspond to a reduction in mortality. This assumption can be tested by studying stage-specific net survival separately depending on screening history.

Estimating stage-specific survival by route to diagnosis is essential to confirm that screening does not result in worse stage-specific survival outcomes and provides a true benefit.^5^ This study aimed to investigate whether survival improvements associated with breast cancer screening are solely attributable to detection at an early stage and whether one could predict future breast cancer mortality using only stage and date of diagnosis. By linking Danish breast screening data with national death records, we estimated expected survival based on screening history, enabling more realistic comparisons than possible without such detailed data.

## Methods

### Study design

We conducted a nationwide register-based cohort study from January 1, 2010 to November 2022 using data on screening attendance before December 31, 2019. This study was a collaboration between King’s College London, the University Research Clinic for Cancer Screening, Randers Regional Hospital, Denmark, and the Department of Biochemistry and Immunology, Lillebaelt Hospital, Denmark.

### Participants

Women were included if they were invited to the Danish Breast Screening Program between January 2010 and December 2019, had no previous breast cancer diagnosis and turned 50 before December 31, 2014 (to allow for at least 8 years of follow-up). Women with a missing date of birth were excluded, although these numbers were small (only 53 women). Women turning 50 before screening implementation entered the study on January 1, 2010, while women turning 50 after this date entered on their 50th birthday. Individuals were followed until they died, emigrated, or until the end of the study period (November 2022). Individuals were right-censored if they emigrated (6182 women [0.8%]) or were alive at the end of follow-up. No participants were left-censored since women with a previous diagnosis were excluded, and information on subsequent diagnoses was available.

### Variables

Demographic variables included a woman’s personal identification number (Central Person Registration (CPR) number), date of birth, Charlson comorbidity index,^6^ study entry date, date of death, vital status (dead/alive), and last follow-up date.

Screening-related variables included the screening date and result (both missing for nonattenders). For route to diagnosis, we operated with 3 categories: (1) screen-detected cancers diagnosed within 6 months of an abnormal screen, (2) symptomatic ever-screened cancers defined as interval cancers diagnosed within 24 months after a normal screen or 6-24 months after an abnormal screen, or symptomatic noninterval cancers among women who were screened previously (>2 years before diagnosis). Finally, we operated with never-screened cancers, defined as cancers diagnosed among screen-eligible women with no screening history. We selected 6 months as the cutoff point for screen-detected cancers following an abnormal screening result.^7^ Consequently, women diagnosed more than 6 months after an abnormal screen, but before their next scheduled screening round, were classified as having interval cancers. This classification reflects the assumption that the eventual cancer diagnosis was likely not the result of organized follow-up within the screening program, but rather due to later symptomatic presentation. In Denmark, opportunistic mammograms are extremely rare; mammograms conducted outside of screening invitation are a result of symptomatic presentation, leading to categorization into either the “never-screened” or “symptomatic ever-screened” groups.

Cancer diagnosis and treatment variables included the presence or absence of a diagnosis, diagnosis date (missing if no diagnosis), route to diagnosis (sporadic, interval, screen-detected), TNM stage (0, I, II, IIa, IIb, III, IIIa, IIIb, IIIc, IV, missing), and corresponding treatment (surgery, treatment without surgery, no treatment). Treatment was categorized into 3 broad groups based on evidence of improved survival in surgically treated stage IV breast cancer (8-10). Danish cancer care dictates timely treatment, so patients who were not too frail likely received chemotherapy or hormonal therapy. Both ductal carcinomas in situ (DCIS) and invasive cases were initially counted, though the main analyses focused on stage-specific survival (I-IV and unknown), effectively excluding DCIS. Stage at diagnosis was defined using clinical information from the Cancer Registry up to December 31, 2020 and pathological information from the Pathology Register from January 2021 onward.

### Data sources

Individual-level data were obtained from 5 high-quality national registries. Information on screening invitations, appointments, and results was retrieved from the Danish Quality Database for Mammography Screening (DKMS).^8^ Cancer diagnosis data were obtained from the Cancer Registry (containing diagnoses up to December 31, 2020)^9^ and the Pathology Register (January 2021-October 3, 2022).^10^^,^^11^ Data on surgical treatment were obtained from both the National Pathology Register^10^^,^^11^ and the National Patient Register.^12^ CPR numbers were used to link each registry with birth, death, and emigration dates from the Civil Registration System.^13^

### Quantitative variables

We used the first recorded diagnosis for women with more than 1 registered breast cancer. For inconsistencies between invasive status and recorded TNM stage, we prioritized stage.

We included treatments administered within 1 year after the registered diagnosis date (taken from the Danish Cancer Registry). We extracted data on resections (pathological breast tissue excisions, microdiscectomies, lactiferous duct excisions, areola or nipple excisions, segmental breast resections, second breast resections, and lumpectomies) and mastectomies (subcutaneous mastectomy with preservation or excision of papilla mammae, total mastectomies, radical mastectomies, second mastectomies, and supernumerary mammary gland and mamimlla excision), including women with these records in our “treated with surgery” category. Women with a treatment recorded which did not include a surgery specification were categorized as being “treated without surgery.” A lack of records within the 2 treatment registers within a year after diagnosis was considered to indicate no treatment.

### Statistical methods

Stage-specific net survival was calculated from excess mortality by subtracting age-specific (all-cause) mortality rates in women without a diagnosis from the observed mortality in women diagnosed with breast cancer.

We calculated mortality (ie, life tables) in women without breast cancer by single year of age from ages 50-79 years and as a single rate for age 80 years and above (due to sparse numbers). In those with breast cancer, we estimated excess mortality rates separately for 0-<2, 2-<5, 5-<10, and 10+ years since diagnosis.

Life tables were generated using Danish data for women without breast cancer who were invited to screening, separately for those previously ever- or never-screened (Figure S1). All women started as never-screened. Anyone who attended screening moved from the never-screened cohort to the ever-screened cohort on their first screening date. Single-year mortality was calculated from the change in the cumulative hazards estimated in this way. Negative excess hazards were replaced by 0.

Net survival was calculated from the excess hazards in the usual way.^14^ Log-transformed 95% confidence intervals (CIs) were calculated using the delta method.^15^ Follow-up was censored at 10 years in women diagnosed after turning 70 years of age, to avoid low counts.

For women diagnosed before the age of 70 years old, we compared never-screened, “symptomatic ever-screened” and screen-detected survival using separate life tables for never-screened and ever-screened women. We also investigated treatment differences by route to diagnosis, focusing on stage IV only due to the notable survival differences observed. After observing that the use of separate life tables for never-screened and ever-screened women was inappropriate with age more than 70 years (Figures S2 and S3), we compared never-screened and ever-screened women using a single life table, regardless of screening status. We separated women below and more than 70 years of age to account for the end of the screening age range, though we are aware that some cancers diagnosed at age 70 may have been screen-detected following a screen before their 70th birthday or just after. However, these women were still categorized as “ever-screened” in the 'above 70 years of age' group, since there would be too few screen-detected cancers in the older age group for separate analyses to be meaningful.

In older women, we also compared survival by comorbidity levels, using the Charlson comorbidity index (Table S1) and (for ever-screened women) by time since last screen.

## Results

### Study population

**Figure 1.**
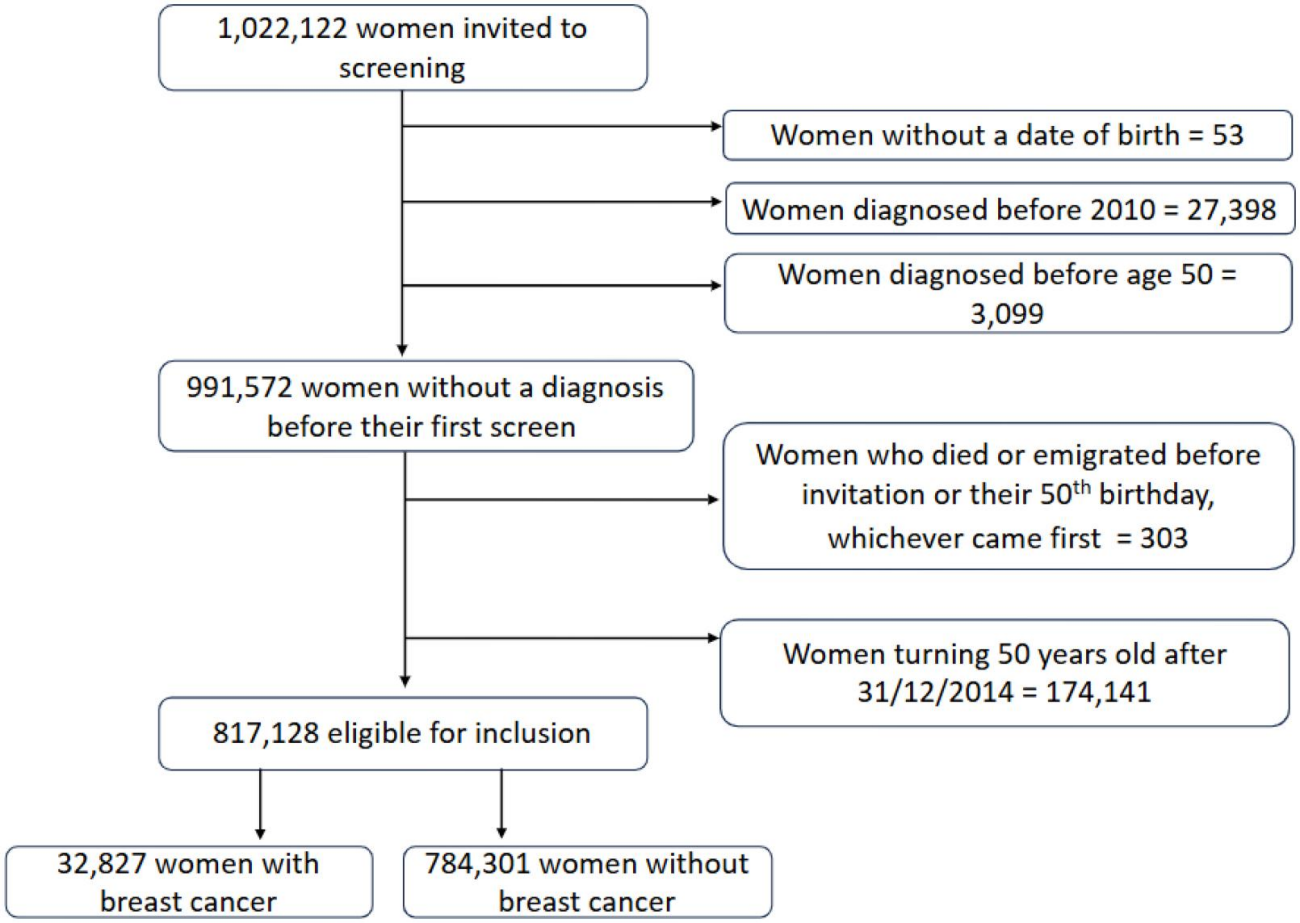
Flowchart of the study population.

Just over 1 million women were invited for screening, 991 572 of whom had no prior breast cancer diagnosis and 817 128 of whom met our inclusion criteria. Of these, 32 827 had a breast cancer diagnosis (arrival at the study cohort is illustrated in Figure 1). Of the cancers, 27 007 (82%) were diagnosed before age 70 and 5820 after; 2991 (9%) of the breast cancers were in situ (stage 0), 14 985 (46%) were stage I, 10 312 (31%) were stage II, and 2787 (8%) were stage III or IV. Stage distribution by group can be found in Table 1. Screen-detected cancers were more likely to be stages 0 and I and less likely to be stage III or IV than nonscreen-detected cancers. Comorbidity severity distribution within the patient cohort was as expected (Table S2).

**Table 1.** Characteristics table for the analysis cohort, presenting the number of Danish women diagnosed with breast cancer between 2010 and 2014, split by age at diagnosis, screening exposure, and stage.

	Women diagnosed before age 70 years (*n* = 27 007)						Women diagnosed after age 70 years (*n* = 5820)				Total	
Total number of patients	4555		7380		15 072		1777		4043			
Stage	Number	% of all stages	Number	% of all stages	Number	% of all stages	Number	% of all stages	Number	% of all stages	Number	%of all stages
0	267	6	492	7	1993	13	53	3	186	5	2991	9
I	1409	31	2911	39	8450	56	557	31	1658	41	14 985	46
II	1770	39	2718	37	3584	24	769	43	1471	36	10 312	31
III	504	11	587	8	506	3	151	8	281	7	2029	6
IV	273	6	188	3	86	1	82	5	129	3	758	2
Unknown	332	7	484	7	453	3	165	9	318	8	1752	5

### Stage-specific net survival

Stage-specific net survival in symptomatic ever-screened women resembled that of never-screened women (Table 2), except for unknown stage, where nonscreen-detected ever-screening demonstrated better survival than never-screened.

**Table 2.** Estimates (with 95% confidence intervals) for 5- and 10-year net survival in women diagnosed with breast cancer before the age of 70 years, by stage and substage.

	Stage	Never-screened	Symptomatic ever-screened	Screen-detected
5-year survival	**I**	**97.6% (96.1% to 99.1%)**	**97.1% (96.2% to 98.1%)**	**99.6% (99.2% to 100.0%)**
	**II**	**93.0% (91.4% to 94.7%)**	**93.3% (92.1% to 94.5%)**	**96.7% (95.9% to 97.5%)**
	IIa	94.7% (93.6% to 97.4%)	94.4% (93.0% to 95.8%)	97.1% (96.2% to 98.0%)
	IIb	89.9% (87.0% to 92.9%)	90.9% (88.5% to 93.3%)	95.3% (93.5% to 97.2%)
	**III**	**76.3% (72.3% to 80.7%)**	**80.7% (77.2% to 84.4%)**	**90.5% (87.6% to 93.5%)**
	IIIa	80.1% (75.1% to 85.5%)	84.1% (80.0% to 88.4%)	93.8% (90.7% to 97.0%)
	IIIb	69.0% (59.0% to 80.7%)	76.8% (66.2% to 89.0%)	82.4% (68.5% to 99.0%)
	IIIc	72.6% (64.1% to 82.3%)	73.3% (65.5% to 82.0%)	84.8% (78.6% to 91.4%)
	**IV**	**32.4% (27.0% to 38.8%)**	**30.9% (24.5% to 38.8%)**	**74.7% (65.5% to 85.1%)**
	**Missing**	**66.6% (61.6% to 72.0%)**	**80.6% (77.0% to 84.4%)**	**96.3% (94.3% to 98.4%)**
10-year survival	**I**	**96.7% (94.3% to 99.1%)**	**94.9% (93.2% to 96.8%)**	**97.0% (96.2% to 97.9%)**
	**II**	**87.8% (85.4% to 90.3%)**	**87.1% (85.0% to 89.2%)**	**92.0% (90.6% to 93.5%)**
	IIa	90.6% (88.4% to 94.3%)	89.2% (86.7% to 91.6%)	93.1% (91.5% to 94.7%)
	IIb	82.5% (78.3% to 87.0%)	82.7% (78.8% to 86.7%)	88.0% (84.8% to 91.4%)
	**III**	**66.8% (61.7% to 72.3%)**	**66.9% (61.9% to 72.4%)**	**70.5% (65.5% to 75.9%)**
	IIIa	73.0% (66.7% to 79.9%)	73.6% (67.8% to 80.0%)	79.5% (73.8% to 85.7%)
	IIIb	59.3% (47.3% to 74.3%)	59.9% (45.6% to 78.7%)	41.7% (25.7% to 67.8%)
	IIIc	57.3% (47.1% to 69.7%)	52.4% (42.3% to 64.7%)	58.2% (48.9% to 69.2%)
	**IV**	**17.1% (12.4% to 23.5%)**	**19.4% (13.0% to 29.0%)**	**61.5% (50.2% to 75.3%)**
	**Missing**	**52.0% (45.1% to 60.0%)**	**76.8% (71.5% to 82.5%)**	**90.8% (85.9% to 95.8%)**

Estimates are presented by screening group: never-screened, symptomatic ever-screened (women with a screening history who had a symptomatic diagnosis) and screen-detected. Rows in bold represent survival estimates for stages, rather than substages, for easier comparison of stage-specific survival differences.

**Figure 2.**
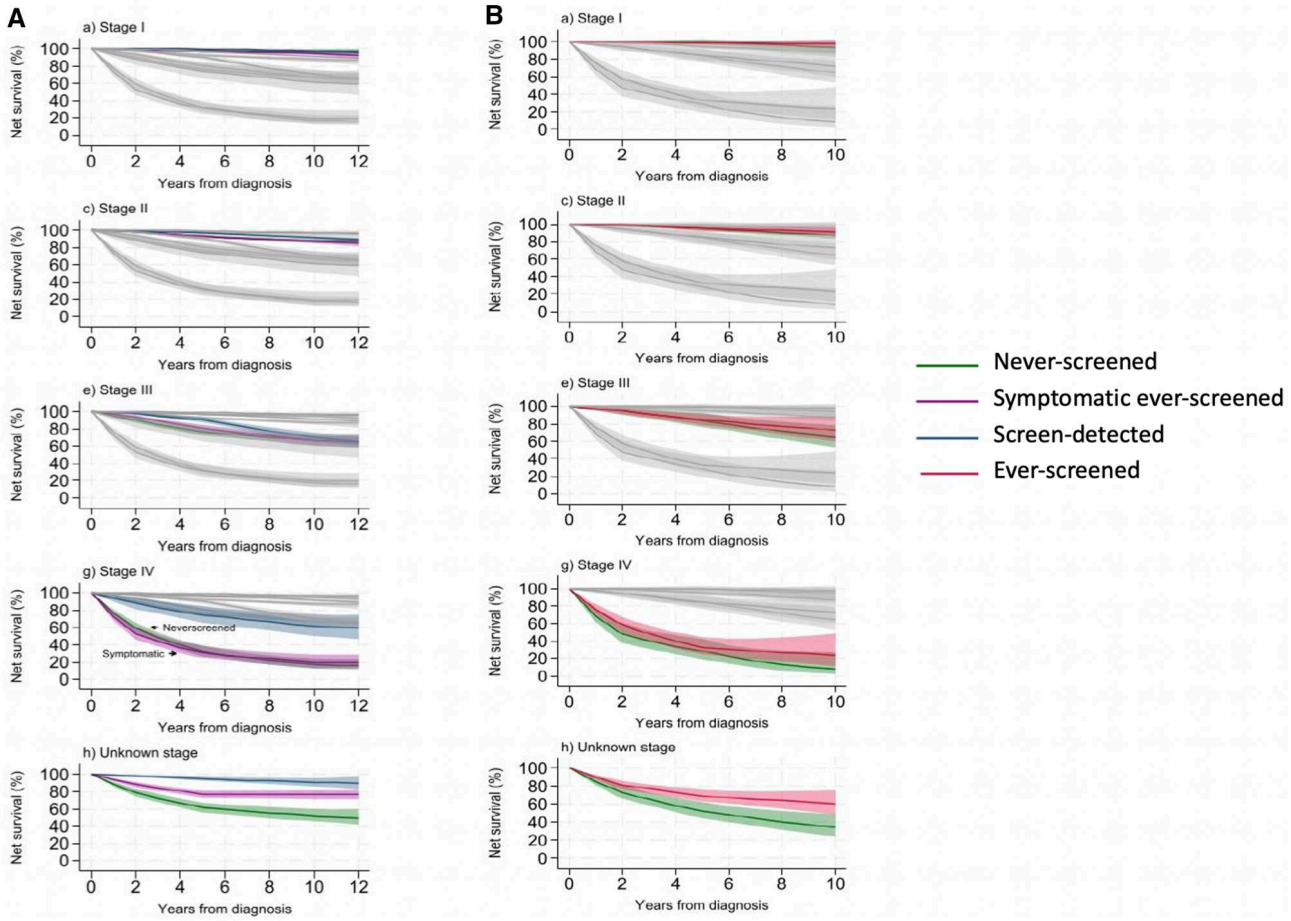
**A)** Net survival, by stage, in women diagnosed with invasive breast cancer before age 70 years (screen-detected women, never-screened women, and "symptomatic ever-screened" women). **B)** Net survival, by stage, in women diagnosed with invasive breast cancer after age 70 years (never-screened women and ever-screened women). Except for unknown stage, plots display curves for all stages, with the stage of interest being highlighted. Solid lines represent survival estimates, while shading represents 95% confidence intervals.

Within stages I, II, and III, estimated net survival was similar regardless of screening history (Figure 2). Numbers at risk are provided in Table S3. Screen-detected women experienced slightly improved stage-specific survival than nonscreen-detected cancers (regardless of screening history), although the differences are too small to perceive in Figure 2.

Large differences were observed for stage IV. Survival in screen-detected stage IV resembled that of nonscreen-detected stage IIIc (Figure S4). Five-year and 10-year net survival in screen-detected stage IV was 75% and 62%, respectively, compared with 32% and 17% in never-screened women (Table 2).

Using a 7-point ordinal scale for stage and substage, screen-detected women appeared to have a 5-year net survival resembling nonscreen-detected women 1-point lower in the scale (Table 2). At 10 years, the survival advantage is less pronounced than a substage shift for stages I-IIIb. The 62% 10-year net survival for screen-detected stage IV compares favorably with that for nonscreen-detected stages IIIb and IIIc cancers (52.4%-59.9%).

Un-staged cancers in never-screened women had survival between that observed for stages III and IV; in ever-screened women, it was between that in stages II and III; and in screen-detected women, it was between stages I and II (Figure 2).

For women more than 70 years, we observed marginally improved stage-specific survival in ever-screened women compared with never-screened women, though not significantly.

### Treatment of stage IV breast cancer

Median net survival of stage IV cancers by treatment and screening history for those diagnosed under the age of 70 years is presented in Table 3. While 4% of stage IV patients received no treatment, all screen-detected metastatic patients received some treatment, with two-thirds having a surgical procedure.

**Table 3.** Treatment outcomes in women diagnosed with a stage IV breast cancer before the age of 70 years, split by screening status.

	All (*n* = 547)	Never-screened (*n* = 273)			Symptomatic ever-screened (*n* = 188)			Screen-detected (*n* = 86)			
	10-year survival (%)	Number	% of stage IV cases	Median survival in years	Number	% of stage IV cases	Median survival in years	Number	% of stage IV cases	Median survival in years	Median survival in years
Surgery within a year after diagnosis (*n* = 170)	59.7 (51.2-69.7)	62	23	6.0 (3.3-11.2)	50	27	4.5 (2.9-7.9)	58	67	7.6 (4.6-10.3)	6.0 (3.4-10.0)
Treated without surgery within a year after diagnosis (*n* = 355)	8.3 (5.2-13.0)	198	73	2.2 (0.9-4.0)	129	69	1.6 (0.6-3.4)	28	33	5.2 (1.7-9.8)	2.0 (0.8-4.0)
No treatment (*n* = 22)	13.5 (6.8-26.8)	13	5	0.1 (0.0 -0.3)	9	5	0.0 (0.0-0.1)	0	0	N/A	0.1 (0.0-0.3)

Only treatments received within 1 year after the diagnosis date (from the Danish Cancer Registry) are included. Median survival for each treatment group and 10-year survival rates are also included, with lower and upper quartiles.

Survival was strongly associated with treatment type. Women receiving surgery had a median survival of 6.0 years (95% CI = 3.4 to 10.0 years), compared with 2.0 (0.8 to 4.0) years for nonsurgical treatment and 0.1 (0.0 to 0.3) years for no treatment. Combining screen-detected and symptomatic ever-screened, treatment-specific median survival times were similar in never-screened and ever-screened women with stage IV cancer. Ignoring screening history, net survival at 10 years was still substantial in those receiving surgery (59.7%) compared with 8.3% and 13.5% for treatment without surgery and no treatment, respectively.

## Discussion

This study examined net survival differences between screen-detected and nonscreen-detected breast cancer populations in Denmark. Screen-detected cases consistently demonstrated superior survival across all stages. Although the survival advantage was modest for stages I-III cancers, there was a 40% difference across 12 years for stage IV breast cancers. We noted a greater likelihood of surgical interventions in stage IV screen-detected patients (two-thirds vs one-quarter for nonscreen-detected cases). Ten-year survival among stage IV cases was 60% in those who received surgery compared with 8% in those treated without surgery. Unknown-stage cancers demonstrated different case mixes by route to diagnosis, and women more than 70 years (all classified as nonscreen-detected) showed no survival differences linked to previous screening attendance.

Net survival is useful for assessing whether breast screening results in a survival benefit.^16^ While net survival does not rely on cause of death, it is subject to lead-time bias.^17^ Survival rates should, therefore, be compared within an individual stage^18^^,^^19^ to ensure that the improved survival associated with earlier stage at diagnosis is realized. The hypothesis that some cancers are inherently “born to be bad” suggests that these cancers typically present at late stages and maintain a poor prognosis even when detected early through screening.^20-22^ This may lead to poorer stage-specific survival in screen-detected cases compared with those diagnosed outside of the screening program.

Our findings indicate a 1-substage survival advantage for screen-detected breast cancers, particularly at advanced stages. For stages I-III, survival differences were minimal. However, screen-detected stage IV cancers had markedly better outcomes, extending to 12 years postdiagnosis, suggesting earlier detection of oligometastatic disease rather than solely lead-time bias.

This superior survival may reflect multiple factors. Higher rates of surgical interventions among these patients indicate a clinical focus on curative intent. Nonscreen-detected patients were not disadvantaged financially, as treatment is free in Denmark, and prior studies show improved survival with surgery in stage IV cases.^23-25^ Therefore, we do not believe that surgery would help nonsurgically treated patients.^26^ Most screen-detected stage IV cancers received surgery, supporting the view that these cases are more often oligometastatic^27-30^ and diagnosed early enough for surgery to be beneficial.

Although some screen-detected cancers may have been symptomatic, these numbers are likely small,^31^ though we did not have access to such data. We also lacked recurrence data; however, if recurrences among screen-detected women were common, we would expect lower early stage survival in this group, which we did not observe. Further, while residual lead-time bias remains a possibility, our 10-year net survival approach helps account for this effect. Improved staging in screen-detected cancers, evidenced by missing stage cases, could inflate survival estimates (“Will Rogers” effect)^32^; however, higher proportions of missing stage in never-screened patients may also stem from higher proportions of un-treated, stage IV cancer. Finally, although coding practice changes may affect stage-specific incidence,^33^ annual stage IV and missing-stage incidence rates in our cohort only slightly increased (Table S4).

These results emphasize that for stages I-III, stage-specific survival may be combined with stage at diagnosis for making mortality predictions. However, survival differences in stage IV screen-detected cases mean that failing to account for detection mode may underestimate the impact of screening in this group. A possible explanation for minimal differences at stages I-III is the relative homogeneity of early stage breast cancer outcomes, whereas stage IV cancers largely vary in metastatic burden, with screening likely detecting less advanced cases, ultimately contributing to the observed survival advantage.

Limited studies have examined survival differences stage-for-stage by screening status. Consistent with our findings, O’Brien et al. reported no significant early stage survival differences after accounting for lead-time bias and other factors.^34^ Contrastingly, a large Oxford study identified lower mortality in screen-detected early stage cancers.^35^ Wishart et al. report similar results to ours, observing a 7%-10% increase in overall 5-year survival for Nottingham Prognostic Index levels “moderate 2” and “poor” (most resembling stage IV).^18^ Other research also supports better outcomes for metastatic patients undergoing surgical treatment, with Harris et al.^23^ reporting significant mortality reductions with surgery, aligning with our observations for stage IV cases.

We identified only 1 other study that utilized similar methodology to ours regarding life table construction, though this was for a prostate cancer screening trial.^36^

Our study compared net survival by screening status while accounting for expected deaths based on screening participation. The use of comprehensive, population-based registers ensured high data quality and allowed robust survival analysis. Constructing life tables specific to the screened population further enhances the accuracy of our findings.

Our main limitation was defining screening implementation as January 1, 2010. Although this marks the completion of national rollout, screening was gradually introduced from 2007, so some women diagnosed in early 2010 were misclassified. We estimate around 500 women were misclassified in each direction, likely causing only minor underestimation of differences between never-screened, symptomatic ever-screened, and screen-detected groups. Other limitations include the observational design, reliance on net survival rather than cause-specific mortality, exclusion of prediagnosis treatments, and limited analysis of nonsurgical treatments. We also lacked detailed sociodemographic and behavioral data, which may have influenced survival differences in the 3 groups. A higher proportion of immigrants—who in Denmark have lower education but lower mortality^37^—among never-screened women could have attenuated the observed differences, though similar survival between symptomatic ever-screened and never-screened women suggests these factors did not fully explain our findings. Finally, the study’s setting within the Danish breast screening program may limit generalizability, though Danish breast cancer survival is comparable to other Western countries.^38^

In conclusion, screen-detected breast cancers show no evidence of worse survival stage-for-stage compared with nonscreen-detected cases. Stage-specific survival is a reasonable estimator for mortality in stages I-III, with minimal bias from screening history. However, for stage IV cancers, one should make allowances for better survival in screen-detected cancers. Whenever breast cancer screening is associated with a reduction in cancers at stages II-IV, it is likely that (with additional follow-up) it will lead to a reduction in breast cancer mortality.

## Supplementary Material

djaf377_Supplementary_Data

## Data Availability

The data underlying this article cannot be shared due to the privacy of Danish individuals. This study analyzed precollected, individual-level data available within 5 Danish registries and did not involve the collection of any new data. Access to data at Statistics Denmark is made available for researchers who are connected to a Danish research institution.
