# Supplementary material for: Improved stage-specific survival in screen-detected breast cancer in Denmark: a cohort study
Source: J Natl Cancer Inst. 2026 Feb 19;118(6):1073–80. doi: 10.1093/jnci/djaf377 (PMC13247330; doi:10.1093/jnci/djaf377)
Supplement: djaf377_Supplementary_Data [file djaf377_supplementary_data.docx]

Table S1: The numbers and percentages of breast cancer patients diagnosed after age 70 within each comorbidity level, split by screening status (never-screened, ever-screened with last screen before age 67 and ever-screened with last screen after age 67). Patients were assigned to a comorbidity level using their Charlson comorbidity index, which measures the extent of concurrent conditions in an individual through the application of weights to diseases, based on one-year mortality hazard ratios.

|  | **Never-screened**  **(n=1777)** | | **Last screened before age 67**  **(n=151)** | | **Last screened after age 67**  **(n=3892)** | |
| --- | --- | --- | --- | --- | --- | --- |
|  | **Number** | **Percentage** | **Number** | **Percentage** | **Number** | **Percentage** |
| **Group 1 (None)** | 1201 | 68% | 90 | 60% | 2606 | 67% |
| **Group 2 (Mild)** | 448 | 25% | 45 | 30% | 1002 | 26% |
| **Group 3 (Moderate)** | 98 | 6% | 9 | 6% | 209 | 5% |
| **Group 4 (Severe)** | 30 | 2% | 7 | 5% | 75 | 2% |
| The Charlson Comorbidity Index predicts 10-year survival based on the presence or absence of 19 chronic conditions. Conditions and age are assigned weights between 1 and 6 based on severity. Groups are calculated as follows: 1 = score 0, 2 = score 1-2, 3 = score 3-4 and 4 = scores >4. | | | | | | |

Table S2: Numbers and percentages of Charlson Comorbidity Index (CCI) scores, separately for women diagnosed before and after age 70. Scores were defined on a woman’s diagnosis date, with scores in group 4 (CCI-score>4) indicating the presence of at least four lifestyle diseases. These include but are not limited to heart disease, respiratory illnesses, cancers, metastases or AIDS.

|  |  | Below 70 years old | Above 70 years old | Total |
| --- | --- | --- | --- | --- |
| None | Number | 21,677 | 3,897 | 25,574 |
|  | Percentage (%) | 80.3 | 67.0 | 77.9 |
| CCI score 1-2 | Number | 5,953 | 4,458 | 1,495 |
|  | Percentage (%) | 16.5 | 25.7 | 18.1 |
| CCI-score 3-4 | Number | 963 | 647 | 316 |
|  | Percentage (%) | 2.4 | 5.4 | 2.9 |
| CCI-score >4 | Number | 337 | 225 | 112 |
|  | Percentage (%) | 0.8 | 1.9 | 1.0 |
| CCI – Charlson Comorbidity index | | | | |

Table S3: Table of numbers of individuals at risk from dying at 0, 2, 5, 8 and 10 years after a breast cancer diagnosis, split by screening group, stage, substage and age group. Women diagnosed under age 70 are split into groups of never-screened, symptomatic ever-screened and screen-detected, whilst women over 70 are split into never-screened and ever-screened cases. For the older age group, numbers at risk are only provided up to 8 years after diagnosis, whilst substages IIIb and c are combined, due to small numbers.

|  |  | **Never-screened (years from diagnosis)** | | | | | **Symptomatic ever-screened (years from diagnosis)** | | | | | | **Screen-detected (years from diagnosis)** | | | | |
| --- | --- | --- | --- | --- | --- | --- | --- | --- | --- | --- | --- | --- | --- | --- | --- | --- | --- |
|  | **Stage** | **0** | **2** | **5** | **8** | **10** | **0** | **2** | **5** | **8** | **10** | | **0** | **2** | **5** | **8** | **10** |
| **Women under age 70** | **I** | 1408 | 1368 | 1218 | 965 | 738 | 2911 | 2848 | 1560 | 806 | 324 | | 8449 | 8357 | 6632 | 4095 | 2311 |
|  | **II** | 1767 | 1695 | 1405 | 1109 | 789 | 2718 | 2641 | 1649 | 885 | 375 | | 3582 | 3527 | 2856 | 1836 | 1111 |
|  | **III** | 504 | 447 | 324 | 245 | 186 | 587 | 547 | 337 | 190 | 82 | | 508 | 492 | 380 | 238 | 136 |
|  | **IV** | 270 | 159 | 70 | 32 | 25 | 187 | 102 | 34 | 13 | 5 | | 86 | 75 | 55 | 36 | 24 |
|  | **IIa** | 1135 | 1098 | 921 | 735 | 519 | 1892 | 1845 | 1129 | 602 | 246 | | 2765 | 2726 | 2210 | 1435 | 860 |
|  | **IIb** | 632 | 597 | 484 | 374 | 270 | 826 | 796 | 520 | 283 | 129 | | 817 | 801 | 646 | 401 | 251 |
|  | **IIIa** | 293 | 264 | 204 | 159 | 123 | 381 | 362 | 230 | 131 | 56 | | 330 | 322 | 225 | 163 | 95 |
|  | **IIIb** | 93 | 79 | 49 | 34 | 24 | 66 | 60 | 36 | 20 | 9 | | 29 | 29 | 21 | 14 | 4 |
|  | **IIIc** | 118 | 104 | 71 | 52 | 39 | 140 | 125 | 71 | 39 | 17 | | 149 | 141 | 104 | 61 | 37 |
| **Women over age 70** |  | **Never-screened (years from diagnosis)** | | | | | **Ever-screened (years from diagnosis)** | | | | | |  | | | | |
|  |  | **0** | **2** | **5** | **8** |  | **0** | **2** | **5** | **8** | |  |  |  |  |  |  |
|  | **I** | 557 | 536 | 392 | 192 |  | 1658 | 1595 | 660 | 175 | |  |  |  |  |  |  |
|  | **II** | 769 | 734 | 523 | 278 |  | 1471 | 1412 | 530 | 128 | |  |  |  |  |  |  |
|  | **III** | 151 | 138 | 90 | 44 |  | 281 | 256 | 95 | 20 | |  |  |  |  |  |  |
|  | **IV** | 80 | 38 | 12 | 3 |  | 129 | 74 | 13 | 3 | |  |  |  |  |  |  |
|  | **IIa** | 504 | 489 | 352 | 193 |  | 1040 | 1005 | 379 | 91 | |  |  |  |  |  |  |
|  | **IIb** | 265 | 245 | 171 | 85 |  | 431 | 407 | 151 | 37 | |  |  |  |  |  |  |
|  | **IIIa** | 100 | 97 | 63 | 31 |  | 193 | 179 | 70 | 17 | |  |  |  |  |  |  |
|  | **IIIb/c** | 51 | 41 | 27 | 13 |  | 88 | 77 | 25 | 3 | |  |  |  |  |  |  |

Table S4: Numbers and percentages of breast cancer cases diagnosed in the study cohort for each calendar year of follow-up, presented by stage at diagnosis.

|  |  | Stage at diagnosis | | | | | | |
| --- | --- | --- | --- | --- | --- | --- | --- | --- |
| Year |  | 0 | 1 | 2 | 3 | 4 | Unknown | Total |
| 2010 | Number | 294 | 1,403 | 1,096 | 261 | 68 | 54 | 3,176 |
|  | Percentage (%) | 9.3 | 44.2 | 34.5 | 8.2 | 2.1 | 1.7 | 100 |
| 2011 | Number | 285 | 1,273 | 943 | 220 | 62 | 41 | 2,824 |
|  | Percentage (%) | 10.1 | 45.1 | 33.4 | 7.8 | 2.2 | 1.5 | 100 |
| 2012 | Number | 252 | 1,283 | 944 | 223 | 82 | 52 | 2,836 |
|  | Percentage (%) | 8.9 | 45.2 | 33.3 | 7.9 | 2.9 | 1.8 | 100 |
| 2013 | Number | 282 | 1,463 | 959 | 198 | 56 | 131 | 3,089 |
|  | Percentage (%) | 9.1 | 47.4 | 31.1 | 6.4 | 1.8 | 4.2 | 100 |
| 2014 | Number | 307 | 1,433 | 979 | 171 | 67 | 179 | 3,136 |
|  | Percentage (%) | 9.8 | 45.7 | 31.2 | 5.5 | 2.1 | 5.7 | 100 |
| 2015 | Number | 286 | 1,462 | 935 | 157 | 51 | 161 | 3,052 |
|  | Percentage (%) | 9.4 | 47.9 | 30.6 | 5.1 | 1.7 | 5.3 | 100 |
| 2016 | Number | 269 | 1,328 | 918 | 163 | 54 | 250 | 2,982 |
|  | Percentage (%) | 9.0 | 44.5 | 30.8 | 5.5 | 1.8 | 8.4 | 100 |
| 2017 | Number | 245 | 1,348 | 908 | 150 | 61 | 254 | 2,966 |
|  | Percentage (%) | 8.3 | 45.5 | 30.6 | 5.1 | 2.1 | 8.6 | 100 |
| 2018 | Number | 239 | 1,481 | 848 | 130 | 85 | 191 | 2,974 |
|  | Percentage (%) | 8.0 | 49.8 | 28.5 | 4.4 | 2.9 | 6.4 | 100 |
| 2019 | Number | 279 | 1,291 | 918 | 184 | 94 | 224 | 2,990 |
|  | Percentage (%) | 9.3 | 43.2 | 30.7 | 6.2 | 3.1 | 7.5 | 100 |
| 2020 | Number | 253 | 1,220 | 860 | 176 | 78 | 215 | 2,802 |
|  | Percentage (%) | 9.0 | 43.5 | 30.7 | 6.3 | 2.8 | 7.7 | 100 |
| Total | Number | 2,991 | 14,985 | 10,308 | 2,033 | 758 | 1,752 | 32,827 |
|  | Percentage (%) | 9.1 | 45.7 | 31.4 | 6.2 | 2.3 | 5.3 | 100 |


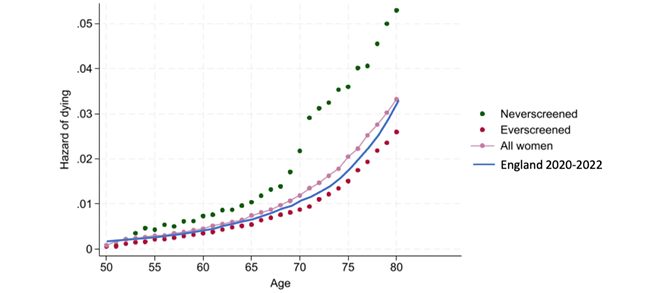


Figure S1: All-cause mortality rates (hazards) in Danish women without a breast cancer diagnosis, by single year of age. Rates are shown separately for never-screened and ever-screened women, as well as for all women without breast cancer regardless of screening status. Published all-cause mortality rates for females in England (2020–2022) are included for comparison, illustrating that the estimated mortality rates in the Danish population are broadly consistent with external data.


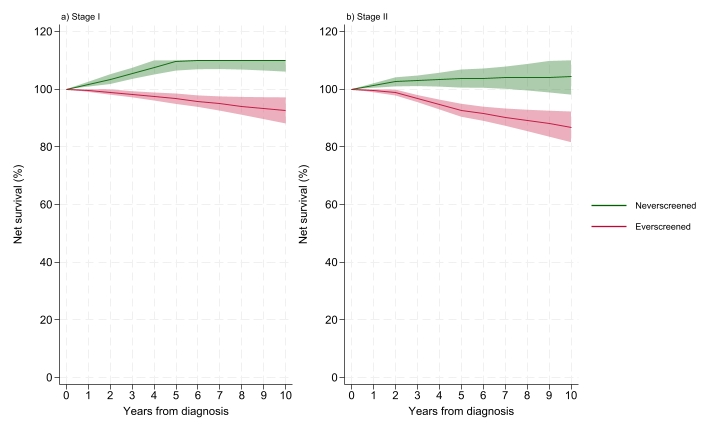


Figure S2: Sensitivity analysis of the methods used to calculate net survival in women diagnosed over age 70. These results are based on the second alternative approach considered (but not utilised) for this age group, in which expected deaths in women with breast cancer were calculated using two sets of all-cause mortality hazards in women without breast cancer. The patient cohort was split into two screening groups only: never-screened and ever-screened. However, these methods produced unexpected findings of improved survival in never-screened women compared to ever-screened women at stages I and II. To investigate whether this approach could still be used to produce appropriate results, the age at diagnosis was restricted a maximum age of 80. Only stage I and II survival rates are presented here as these were the affected stages


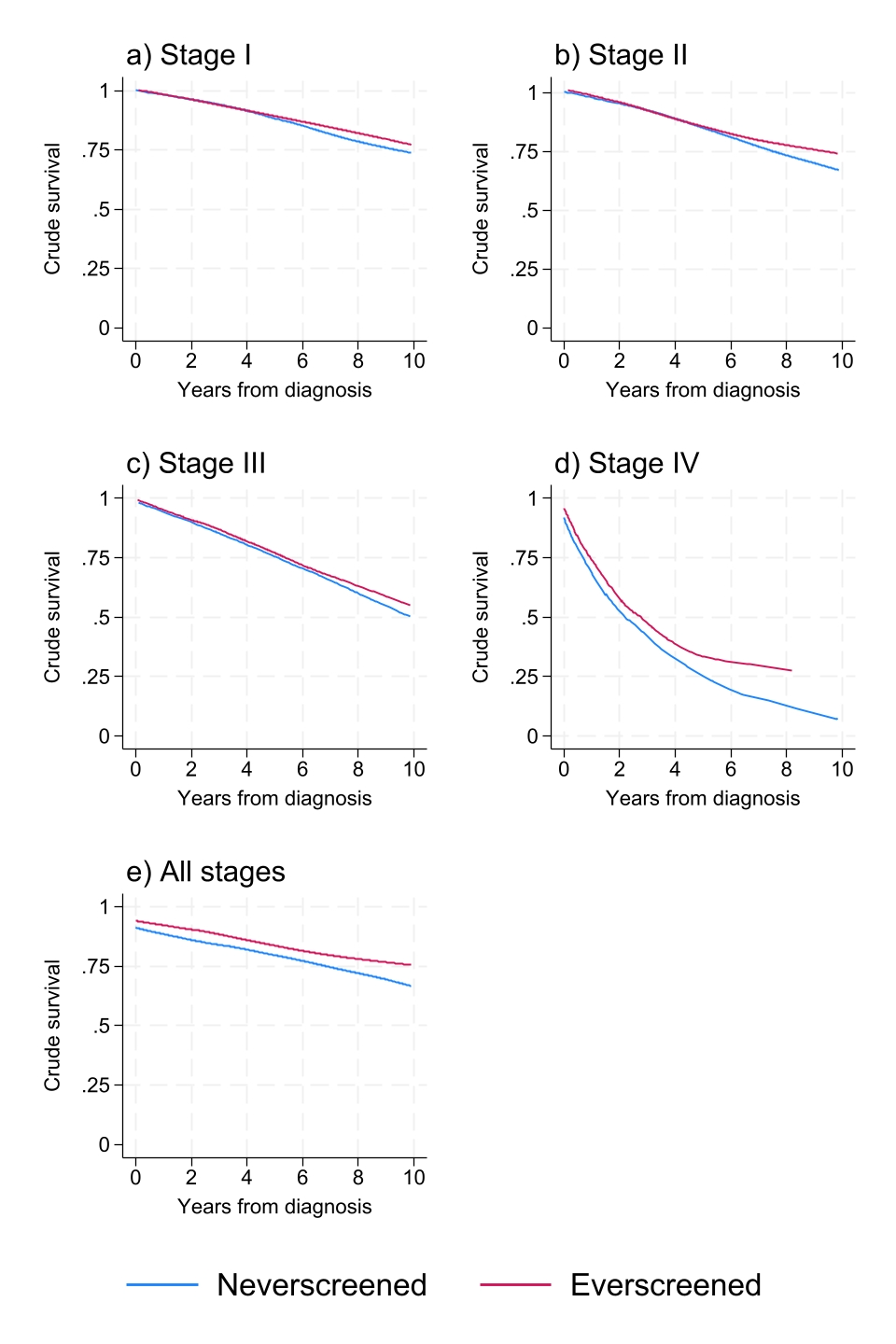


Figure S3: Comparison of crude survival in never-screened and ever-screened women diagnosed over age 70, split by stage at diagnosis. Plot e) presents the crude survival comparison for all stages combined (including unknown stage). Kaplan Meier curves have been smoothed here using the ‘lowess’ function in Stata for data protection purposes.


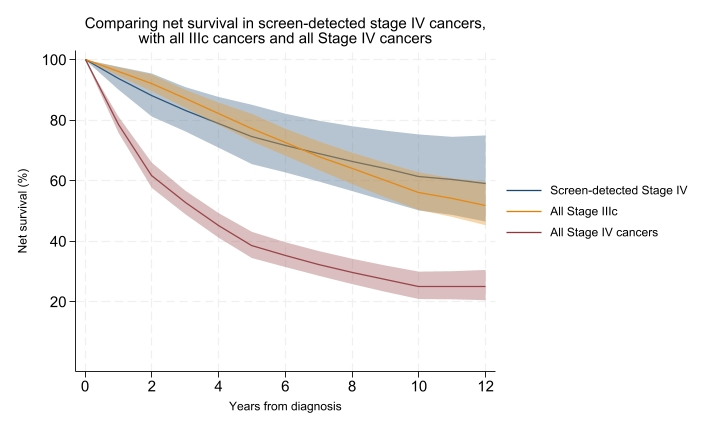


Figure S4: Investigation into stage IV screen-detected cancers. Survival estimates for screen-detected stage IV cancers are plotted with survival rates for stage IIIc cancers and stage IV cancers in all screening groups combined. Solid lines represent survival estimates, whilst shading represents the 95% confidence interval around the estimate. Net survival was calculated based on excess deaths in women with breast cancer. Expected deaths were estimated using separate life tables for screening status, with expected deaths in never-screened women utilising all-cause mortality hazards in never-screened women without breast cancer and expected deaths in the screen-detected and ‘symptomatic ever-screened’ groups being calculated utilising all-cause mortality hazards in ever-screened women without breast cancer.
